# Development, Characterization, and Optimization of Protein Level in Date Bars Using Response Surface Methodology

**DOI:** 10.1100/2012/518702

**Published:** 2012-06-18

**Authors:** Muhammad Nadeem, Salim-ur-Rehman, Faqir Muhammad Anjum, Mian Anjum Murtaza, Ghulam Mueen-ud-Din

**Affiliations:** ^1^Institute of Food Science and Nutrition, University of Sargodha, Sargodha, Pakistan; ^2^National Institute of Food Science and Technology, University of Agriculture, Faisalabad, Pakistan

## Abstract

This project was designed to produce a nourishing date bar with commercial value especially for school going children to meet their body development requirements. Protein level of date bars was optimized using response surface methodology (RSM). Economical and underutilized sources, that is, whey protein concentrate and vetch protein isolates, were explored for protein supplementation. Fourteen date bar treatments were produced using a central composite design (CCD) with 2 variables and 3 levels for each variable. Date bars were then analyzed for nutritional profile. Proximate composition revealed that addition of whey protein concentrate and vetch protein isolates improved the nutritional profile of date bars. Protein level, texture, and taste were considerably improved by incorporating 6.05% whey protein concentrate and 4.35% vetch protein isolates in date bar without affecting any sensory characteristics during storage. Response surface methodology was observed as an economical and effective tool to optimize the ingredient level and to discriminate the interactive effects of independent variables.

## 1. Introduction

The food bars are snacks of good sensory and nutritional characteristics due to their high carbohydrates, proteins, lipids, and minerals contents. Snack foods such as potato chips, extruded products, chocolates bars available in market cannot meet the requirement of balanced diet [1]. These are unhealthy offerings for the consumers especially school going children. Increasing demand from consumers for nutritious snacks, has provoked the food manufacturers to develop food bars that provide nutrition and convenience [2].

School-going children need nutritious foods due to their enhance body development requirements. Food consumed by them should be rich in vitamins, minerals and balanced regarding major nutrients like carbohydrates, proteins and fats. The options available for the children to buy wholesome and nourishing food products are very limited. This gap needs to be filled by developing products that conform to emerging trends of nutraceutical and functional foods [1]. The products that are developed by utilizing dried fruit, processed legumes, and cereals along with nuts would be an attractive snack food for the school going children and for those people working outside their homes and are becoming more dependent on snacks for the supply of part of their daily nutritional requirements [3]. At the moment, the imported fruit bars are available at super stores only in the big cities. Some popular brands are Kellog*ʼ*s Nutri Grain, Nature Velley, and so forth. The market price for these bars is exorbitant and ranged from rupees 85–130 per 35–45 g bar. This price is out of reach for target children (low and middle income families). In principle, the cost of indigenously developed product should be below rupees 10. which will suit the target consumer. Quality and price are key factor for the development of a competitive product. To achieve this objective, economical and underutilized food sources with good nutritional value should be explored. Dates and Indian vetch (*Lathyrus sativus *L.) are good options in this regard as these are abundantly produced but are underutilized.

Date flesh contains substantial amount of carbohydrates (73.5%) along with ash (1.5%), protein (2.3%), lipids (0.2%), vitamins, and fifteen mineral elements [4]. The polysaccharides from date fruit have been used as a functional constituent and provide bioactive compounds in the formulation of drugs [5]. Dates and dried fruit have high concentration of polyphenols with excellent nutritional value that enrich lipoprotein in plasma and protect it from oxidation [6]. These have also been identified as having antioxidant and antimutagenic properties and help in controlling cardiovascular diseases [7].

Similarly, among the grains, corn has high total antioxidant activity followed by wheat, oat, and rice [8]. Whole grains and legumes are also rich sources of protein and dietary fiber. Due to their health benefits, these food stuffs have lured the scientists and technologists for the development of functional foods which is an emerging trend in the new millennium [9]. Indian vetch (*Lathyrus sativus *L.) is one of the cheapest legumes rather least investigated potential source of protein. It is high in good quality protein (28.70 g/100 g) and lysine content with agreeable taste, so it can be used in innovative food product developments. The elimination of antinutritional and toxic factors from said legume makes it a good candidate for supplementation in wheat flour [10]. Its flour can be utilized in different products after detoxification for enrichment in bread [11], chapatti [12], doughnuts [13], and pizza cheese [14]. The primary source of legume protein for supplementation can be utilized in various forms such as flour, concentrates, isolates, or textured vegetables protein [15, 16].

Dates have an edge over other sweet confections, as they not only give natural sugars, sucrose, and fructose but also have an excellent amount of dietary fibers, especially, when they are blended with cereals and legumes [5, 17, 18]. The high moisture content of fresh dates may be absorbed by cereal and legume flours, thus providing suitable matrix to date bar and thereby, boost its storability [19, 20]. Moreover, nutritional properties of dates, nuts, cereals, and legumes may be complementary to each other. Vital nutritional attributes like vitamins and minerals have good bioavailability in natural forms as compared to the conventional processed products.

Response surface methodology (RSM) is reported to be an effective measure for optimizing a process, when the independent variables, for example, protein sources, are hypothesized to possess a sovereign or cumulative effect on the desired responses [21]. Considering the aforementioned essentials, this project was designed to produce a nourishing date bar with commercial value. The present project was planned to assess the suitability of vetch protein isolate and whey protein supplementation in date bar by applying physicochemical tests and to optimize the protein level of date bars by using RSM.

## 2. Materials and Methods

### 2.1. Procurement of Raw Materials

Commercially available dates (Karblain), roasted gram and corn, peanuts, almonds, whey protein concentrate and Indian vetch, common salt, and cardamom were purchased from local market of Faisalabad. Analytical grade chemicals were purchased from Sigma Aldrich (Seelze, Germany) and Lab-Scan (Dublin, Ireland) available in the local market.

### 2.2. Pretreatment of Raw Materials

Dates were pitted, washed, and dried. Pitted dates were steamed for 3 minutes. These were then dried. Peanuts and almonds were shelled, skin removed, and crushed to form grits. Roasted corn and gram were ground to form flour. Butylated hydroxytoluene (BHT) and potassium sorbate were also ground separately with Merlin classic machine to fine powder.

### 2.3. Development of Bars

**Figure 1:**
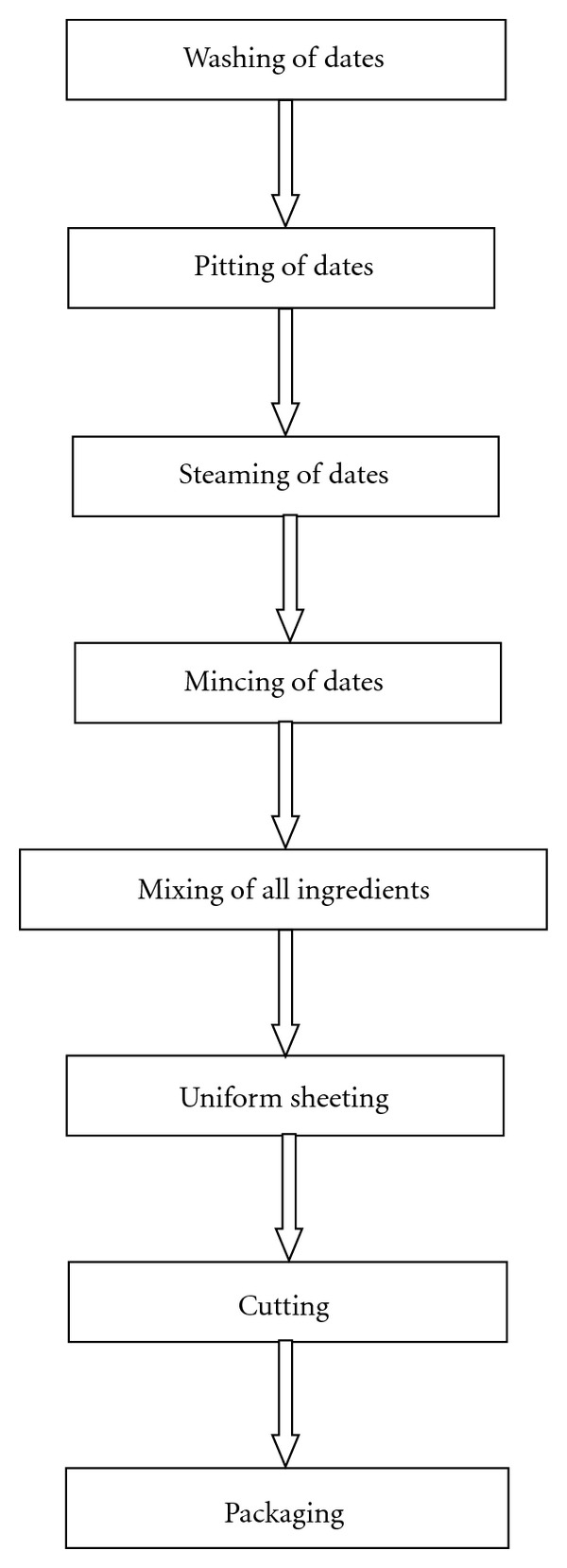
Process flow chart for date bars.

After preparation of raw materials, dates were passed through mincing machine to make paste. Other ingredients (roasted gram flour and corn flour, peanuts, almonds, whey protein concentrate and vetch protein isolates, common salt, cardamom, potassium sorbate, and butylated hydroxytoluene) were mixed thoroughly to distribute uniformly and to make a blend (Table 1). After mixing, sheeting was done, which was cut into bars of 2.5 cm width, 1 cm height, and 7 cm in length. Each bar of approximately 25 g was packed individually in aluminum foil. The quantity of date paste, roasted gram flour and corn flour, peanuts, almonds, common salt, cardamom, potassium sorbate, and butylated hydroxytoluene remained constant except two variables, that is, whey protein concentrate and vetch protein isolate in different proportions (Tables 2 and 3) according to the model created by applying RSM, and control bars were prepared without the addition of protein isolates. The process flow chart is given as follows (Figure 1).

### 2.4. Experimental Design for Protein Level Optimization

RSM was used to optimize the levels of independent variables, that is, vetch protein isolate and whey protein concentrate and their effect on dependent variables. In this study, a response surface box Behnken design was used. Maximum and minimum levels of independent variables were searched out by conducting early trials.

Fourteen date bar treatments were created using response surface design with 2 variables having 3 levels. Total 9 different formulations were produced and runs 4, 5, 8, 9, and 13 correspond to centre point replicates. The complete experiment design for coded and actual levels is presented in Table 3.

### 2.5. Physicochemical Analysis of Date Bars

Date bars were stored at ambient temperature (25 ± 5°C). During storage period, these bars were evaluated for physico-chemical characteristics. 

#### 2.5.1. Texture Analysis

**Figure 2:**
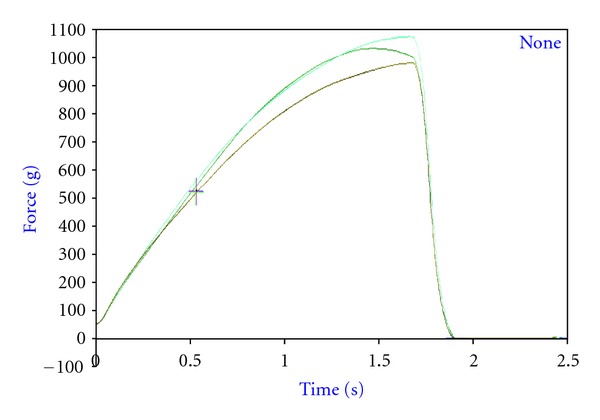
Representative graph of bend test of date bars.

Texture of date bars was determined at different storage intervals according to the method as described by researchers [22] with some modifications by using a texture analyzer (Model TA-XT2.Plus.Stable Microsystems, Surrey, UK) with 5 kg load cell. The Texture Expert program version 4.0.9.0 was used for data analysis. Textural determinations (hardness and fracturability) were made by using a 3 points bending rig (HDP/3PB) for a bend test (Table 4). The bars were bent in order to check different structural characteristics present inside or on the surface. Samples for bending were placed centrally under the 3 points bending rig secured on heavy duty platform (HDP/90). Both the load cell and probes were calibrated before test. Hardness and fracturability measurement of samples by bending involved plotting force (g) and distance (mm) versus time (second). The maximum force (g) was used as an index of hardness (firmness) and distance (mm) as fracturability for the bend test (Figure 2). 

#### 2.5.2. Proximate Analysis

Proximate composition such as moisture, ash, crude protein, crude fat, and crude fiber of date bars was determined and expressed on dry-matter basis [23]. 

#### 2.5.3. Sensory Analysis

Taste of date bars were evaluated at room temperature (i.e., 25 ± 5°C) in a sensory evaluation laboratory by a penal of ten untrained judges on 9-point Hedonic Scale [24].

### 2.6. Statistical Analysis

Results were statistically analyzed by using analysis of variance technique. Level of significance within means was calculated by using the Duncan Multiple Range Test [25]. Minitab (ver. 14.1) statistical software (Minitab Inc., PA, USA) was used for optimization studies. 

## 3. Results

Date bars were developed using selected raw materials and protein levels were optimized by applying RSM. Bars, thus, prepared were analyzed for their physicochemical properties. 

### 3.1. Physicochemical Analysis of Date Bars

#### 3.1.1. Proximate Composition of Date Bars

Proximate analysis includes determination of moisture, crude protein, crude fat, crude fiber, ash, and NFE of date bar samples. The means regarding moisture, crude protein, crude fat, crude fiber, ash, and NFE are given in Table 5. The moisture content of date bar sample ranges from 15.56 ± 0.02 to 18.70 ± 0.02%, crude protein 7.41 ± 0.01 to 14.96 ± 0.01%, crude fat 5.55 ± 0.02 to 8.37 ± 0.01%, crude fiber 3.58 ± 0.01 to 3.91 ± 0.02%, ash 2.30 ± 0.01 to 2.91 ± 0.02%, and NFE 70.85 ± 0.02 to 81.12 ± 0.07%. There was a significantly increasing trend in crude protein, crude fat and ash content, with the addition of whey protein concentrate and vetch protein isolate. Minimum crude protein, crude fat and ash contents were recorded in control bar (*T*_0_) while maximum crude protein content was recorded in *T*_6_ (14.96 ± 0.01%) and crude fat and ash content in *T*_7_ (8.37 ± 0.01%, 2.91 ± 0.02%, resp.). 

### 3.2. Optimization of Protein Levels in Date Bars Using RSM

Dates provide appreciable amount of carbohydrates and other nutrients but are deficient in protein. In order to improve the protein level in date bars, economical and underutilized protein sources have been explored. The date bars were prepared by using the best formulation, incorporating whey protein concentrate and vetch protein isolate at variable levels. RSM was applied to estimate the responses of independent variables that is, whey protein concentrate (*X*) and vetch protein isolate (*Y*) during storage. Second-order polynomial model was fitted for independent variables. The regression equations and coefficients were determined by using multiple regression analysis of storage's data regarding different parameters. 

#### 3.2.1. Hardness (Firmness) of Date Bars

**Figure 3:**
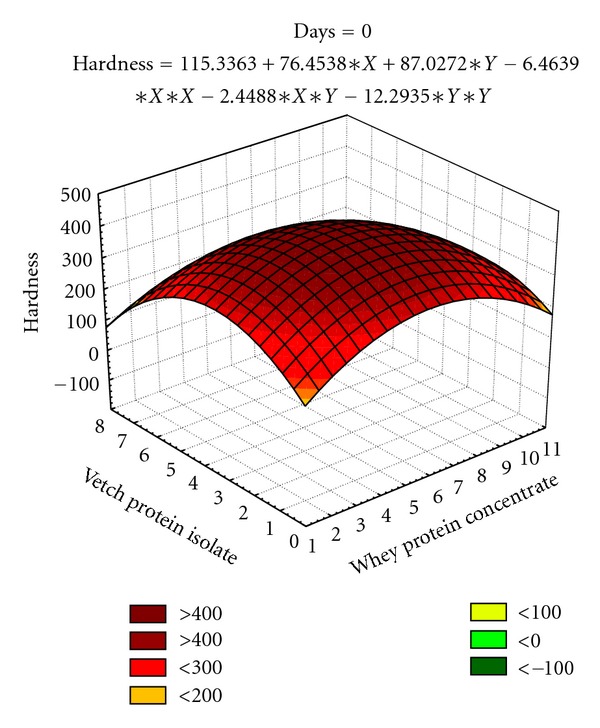
Effect of independent variables (*X*, *Y*) on hardness (firmness) in date bars during storage (at 0 day).

**Figure 4:**
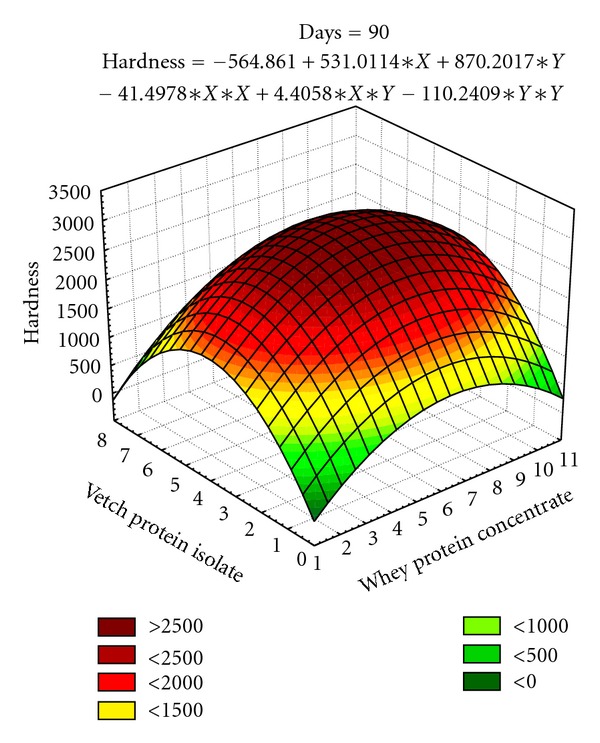
Effect of independent variables (*X*, *Y*) on hardness (firmness) in date bars during storage (at 90 days).

The responses for hardness from central composite design (CCD) were fitted with second order polynomial equations (Table 6). The statistical analysis by applying analysis of variance technique to the full regression of model indicated nonsignificant effect (*P* > 0.05) of variables. However, linear terms of variable (*X*) are observed to negatively change the hardness of bars at all storage intervals, whereas quadratic terms of vetch protein isolate (*Y*) have a positive effect. The interaction of these two terms (*XY*) was found negative over all storage intervals. The coefficients of determination (90.6%) assured that models are adequately fitted. Both independent variables contribute toward increase in firmness, that is, upto 2468.56 g in date bars at 0 to 90 days storage intervals (Figures 3 and 4). 

#### 3.2.2. Fracturability of Date Bars

**Figure 5:**
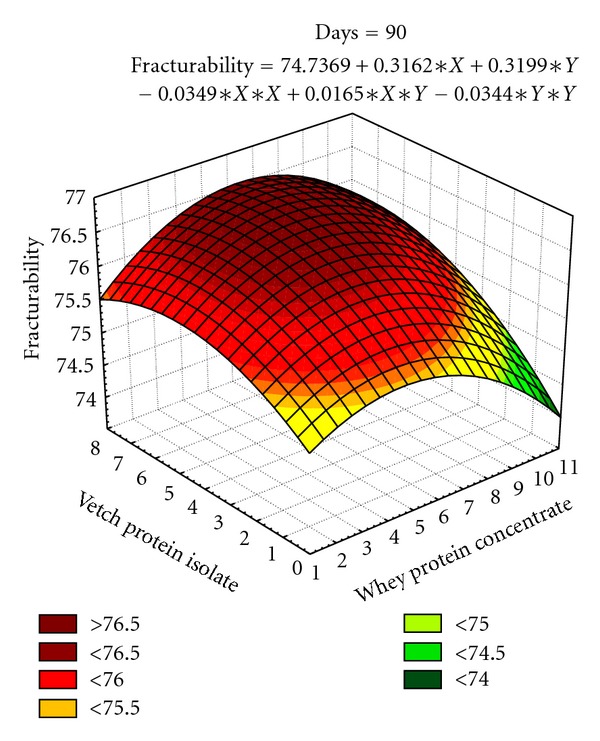
Effect of independent variables (*X*, *Y*) on fracturability in date bars during storage (at 90 days).

The models were developed for fracturability of date bars as affected by independent variables during 90 days storage (Table 7). The effect of linear terms of *X* and *Y* are statistically significant (*P* < 0.05) for fracturability of date bars during storage. The *X* ^2^ quadratic terms are found significant at 15, 45, 60, 75, and 90 days storage intervals whereas the quadratic terms for *Y* ^2^ are found significant at 15, 45, and 60 days. The interaction of two variables (*XY*) shows nonsignificant effect during at all storage intervals. The coefficients of determination (96.5%) assured that models are well fitted. The independent variables contribute towards increase in fracturability in date bars at 0 to 90 days storage intervals (Figure 5). 

#### 3.2.3. Taste of Date Bars

**Figure 6:**
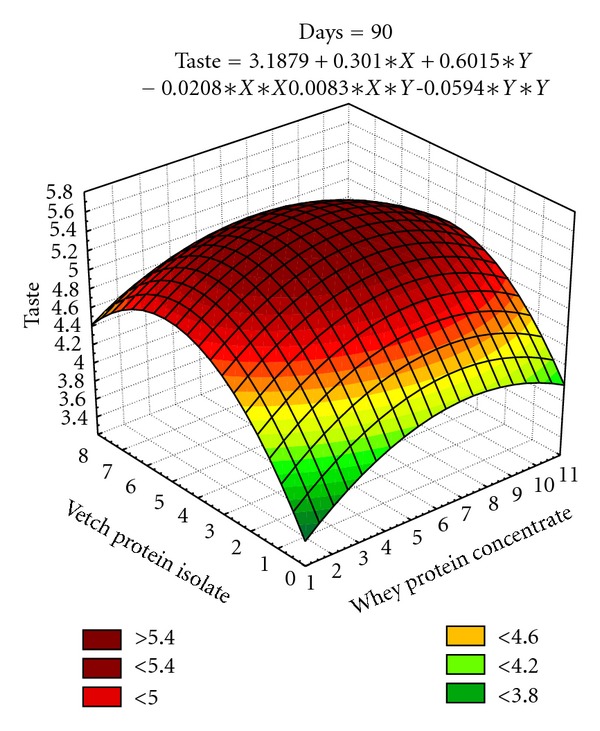
Effect of independent variables (*X*, *Y*) on taste in date bars during storage (at 90 days).

The coefficients of determination (*R* ^2^) for these models (*R* ^2^ = 89.9%, 95.0%, 98.8%, 89.4%, 95.2%, 94.2%, and 89.6% resp.) exhibit the adequacy of models and showed that it covers more variability in data (Table 8). The effect of linear terms of *X* and *Y* for taste of date bars is significant during storage. The *X* ^2^ and *Y* ^2^ quadratic terms are also found significant during storage interval of 90 days. The interaction of two variables (*XY*) shows significant effect on taste at 15 and 30 days of storage intervals. Independent variables (whey protein concentrate and vetch Protein isolate, that is, 5.75% and 4.36%, resp.) have well contributed towards achieving good score for taste of date bars. The scores for taste decline during 90 days storage intervals. However, during the entire storage period, the taste of date bars is found acceptable (Figure 6).

## 4. Discussion

As the dates are not a good source of protein; so, whey protein and vetch protein isolate has been used for the development of date bars. Addition of whey protein concentrate and vetch protein isolate in appropriate proportions has improved nutritional status as well as physical and chemical properties of date bars. The protein content increased to 7.55% in date bars. Similar results were observed by some other research workers who observed an increase in protein level in date bars from 10.7 to 12.1% with the addition of soy protein isolate, single-cell proteins, almonds, and skim milk powder. Moreover, it has improved the chemical scores of essential amino acids in date bars [26, 27]. The protein content of bars can also be increased by fortification of peanut flour, soy flour [28], mesquite cotyledon [1, 29, 30], and black and red beans [31]. Although, fortification of date bars with these sources increases protein, fiber, and ash contents and it also improves minerals such as Ca, Mg, Na, K, P, Zn, and essential amino acids without affecting their sensory acceptability. In the present research work, ash content increases to the tune of 0.60% with the addition of whey protein concentrate. 

Fat is an important constituent of bars which does not only provide energy but also increases the palatability. Moreover, it acts as binder along with sweeteners in agglutination of the ingredients of food bar which are responsible to impart firmness and compactness to texture of food bars [32]. During this study, fat content has been increased in the date bars (2.82%) with the supplementation of whey protein concentrate. 

Optimization of ingredients in the food's formulation is necessary for the development of a product. The optimization of protein and fat levels can also optimize level of carbohydrates of food bars [33]. A candy bar fortified with soy protein contained 58.7% carbohydrates, 12.4% protein, and 9% fat [29]. Similarly, the addition of legume flours increases protein, fat, fiber, ash, minerals, and vitamins in chocolate bars [34]. In the present scanario, high-energy fortified date bars have been developed with an excellent profile of protein, carbohydrate, and fat. These date bars provide good amount of nutrients that can meet the terms of daily nutritional requirements an individual [35]. These can be incorporated in manu of school going children, working people, sportmen, emergency situation, as snack food and nutritional servings. The soy-based candy bar containing 14% protein, 22% fat, and 65% carbohydrates could provide 375.2 kcal/100 g [29]. 

In optimization study, the effect of levels of independent variables; protein sources of dependent variables such as hardness, fracturability, and taste for date bars was optimized. The second-order polynomial models were fitted for independent variables:

(1) $$Z=\beta 0+\beta 1X+\beta 2Y+\beta 11X^{2}+\beta 22Y^{2}+\beta 12XY.$$

In this equation: *Z* = dependent variable to be measured, *β*0 = regression coefficient for treatment effect, *β*1 = regression coefficient for *X*, *β*2 = regression coefficient for *Y*, *X* = coded level of whey protein concentrate, *Y* = coded level of vetch protein isolate. 

A number of techniques is available to find out the best levels of input variables, which in turn optimize their responses [36]. The most straight forward way to undertake is to draw the surface or contour plots of the fitted models. In this study, during data analysis, surface plots were drawn with the help of computer software, Statistica. Three levels, each of whey protein concentrate and vetch protein isolate at different rates were used. The levels were coded as −1, 0 and +1. The relationship between coded (*X*) and experimental levels (*x*) of whey protein concentrate and vetch protein isolate is given as

(2) $$X_{1}=\frac{x_{1}-6}{6},\;\;X_{2}=\frac{x_{2}-4}{4}.$$

Whereas *X*_1_ and *X*_2_ are the coded values for whey protein concentrate and vetch protein isolate, respectively. RSM is employed to check the worth of many factors and their complex interaction through multiple regression analysis [37]. This methodology is now gaining significance in food research studies by optimizing the ingredients level [38], composite flours [39], product improvers [40], and process conditions for product development like temperature, pressure, humidity, pH, and so forth [41, 42]. To optimize the protein levels in date bars, response optimizing function of statistical program Minitab (ver. 14.1) was used. For the optimization process, maximum taste and firm texture were targeted. 

The texture in terms of hardness and fracturability is a feature of prime importance in date bar quality parameters. The surface plots (Figures 3–5) depicted that the maximum hardness (2887.31 g) and fracturability (74.70 mm) were achieved by adding 5.39% and 6.89% whey protein concentrate and 3.69% and 4.24% vetch protein isolate, respectively. In a previous study, response surface methodology was applied to optimize the baking parameters of chapatti, that is, thickness, baking time, and temperature of chapatti. It was found that thickness of chapatti had negative effect on hardness, and cohesiveness, whereas baking time and temperature had positive effects on the hardness and chewiness of the chapatties [43]. Similar results were also observed by researchers working on different types of bars. The bars containing whey protein isolate has shown soft texture throughout storage period which might be due to formation of continuous matrix of protein and sugars, whereas texture became hard in case of bars prepared with calcium caseinate which might happened due to the migration of water molecule from protein towards sugars after 10–18 h of preparation [44]. 

The proteins from different sources may not behave like protein-fortified food bars. Similarly, mango bars with soy protein has higher hardness and springiness. The bars which were fortified with coconut powder have relatively less hardness which might be due to less protein content [45]. Protein bars containing added protein, fat, sugars, and minimum amount of water (water activity in the range of 6.0–6.5) indicated that fracturability force increased and continued with passage of time. During this period, rate of chemical reaction might be decreased and protein particles have crowded together resulting in precipitation of soluble protein due to moisture migration. These observations suggested that the role of chemical reaction is less as compared to variation in microstructure caused by moisture migration in hardening of protein bars [46]. 

The functions of added protein are to keep the ingredients of snack bars intact, set the structure, increase the strength, and contribute to water holding capacity and Maillard browning. The whey protein has considerable viscosity, gel strength, and water holding properties which may contribute to bar firmness during shelf life [47–49]. Moreover, the increase in firmness of bars might be due to the migration of moisture between the carbohydrates (such as starches, pectins, sugars, and maltodextrin) and the proteins [48]. 

Figure 6 depicts 5.5 as maximum score for the taste of date bars at 90 days storage. This value has been selected as target value for taste attribute. The optimized levels for two variables were found as 6.37% (*X*) and 4.64% (*Y*). When these values are put in regression model for taste at 90 days, the calculated value attained is 5.5 as well. 

It is obvious from the results that each aspect of independent variables suggests different optimized levels. The response optimization function of Minitab program was commissioned to reach a cumulative result. For the target values of taste and texture, the optimized variable levels were as follows. 

whey protein concentrate (*X*) = 6.05% vetch protein isolate (*Y*) = 4.35%. 

## 5. Conclusion

Physico-chemical properties and sensory characteristics for date bars were evaluated to assess the suitability of supplementation of protein from two different sources such as plant source (vetch protein isolate) and animal source (whey protein concentrate). The results for proximate composition revealed that addition of whey protein concentrate and vetch protein isolates has significantly elevated the nutritional status of date bars. It has been found that the protein level could considerably be improved by incorporating 6.05% whey protein concentrate and 4.35% vetch protein isolates in date bar without affecting any sensory characteristics (taste) during storage. Response surface methodology was observed as the best tool to discriminate the interactive effects of independent variables. 

These date bars can be commercialized and become a source of foreign exchange. The cost of production of these bars is given in Table 9. The studies revealed that the potential exists to produce an economical date bars. The outcomes derived from present research work would be supportive for the scientists, researchers and stakeholders dealing with food for better understanding of storage stability of date bars.

## Figures and Tables

**Table 1:** Date bars formulation.

Ingredients	Quantity
Date paste	100 g
Composite flour	20 g
Peanuts	10 g
Almonds	10 g
Whey protein concentrate	As per Table 3
Vetch protein isolate	As per Table 3
Salt	0.5 g
Cardamom	1 g
Butylated hydroxytoluene (BHT)	0.002%

**Table 2:** Level of independent variables (%).

Level of independent variable (%)	−1.0	0.0	1.0
Whey protein concentrate	3	6	9
Vetch protein isolate	2	4	6

**Table 3:** Experimental design for date bars formulation (independent variables).

Sr. number	Standard order	Run order	Pt type	Blocks	Whey protein Concentrate (%)	Vetch protein isolate (%)
1	1	1	1	1	3.00	2.00
2	2	2	1	1	9.00	2.00
3	5	3	0	1	6.00	4.00
4	6	4	0	1	6.00	4.00
5	7	5	0	1	6.00	4.00
6	3	6	1	1	3.00	6.00
7	4	7	1	1	9.00	6.00
8	13	8	0	2	6.00	4.00
9	14	9	0	2	6.00	4.00
10	11	10	−1	2	6.00	6.8284
11	9	11	−1	2	10.2426	4.00
12	8	12	−1	2	1.7574	4.00
13	12	13	0	2	6.00	4.00
14	10	14	−1	2	6.00	1.1715

**Table 4:** TA-XT2 settings for comparison of hardness and fracturability of date bars by bend test with 3 points bend rig.

Mode	Measure force in compression
Option	Return To Start
Pretest speed	1.0 mm/s
Test speed	3.0 mm/s
Posttest speed	10.0 mm/s
Distance	5 mm
Trigger force	Auto −50 g
Tare mode	Auto
Data acquisition rate	500 pps

**Table 5:** Mean values for moisture, crude protein, crude fat, crude fiber, ash, and NFE in date bars (%)*.

Treatment	Moisture	Crude protein	Crude fat	Crude fiber	Ash	NFE
*T*_0_	15.56 ± 0.02j	7.41 ± 0.01j	5.55 ± 0.02j	3.58 ± 0.01ef	2.30 ± 0.01g	81.12 ± 0.07a
*T*_1_	16.34 ± 0.01h	9.80 ± 0.02h	6.37 ± 0.02g	3.65 ± 0.02de	2.64 ± 0.02f	77.53 ± 0.01b
*T*_2_	16.63 ± 0.01g	10.49 ± 0.01g	8.03 ± 0.02b	3.84 ± 0.02ab	2.84 ± 0.01abc	74.79 ± 0.02e
*T*_3_	17.23 ± 0.01e	12.16 ± 0.01e	7.20 ± 0.02e	3.74 ± 0.01c	2.80 ± 0.02bcd	74.09 ± 0.01f
*T*_4_	18.20 ± 0.01c	13.76 ± 0.01c	6.16 ± 0.02h	3.56 ± 0.02f	2.66 ± 0.02ef	73.85 ± 0.05g
*T*_5_	18.42 ± 0.01b	14.48 ± 0.01b	7.87 ± 0.01c	3.91 ± 0.02a	2.87 ± 0.02ab	70.85 ± 0.02j
*T*_6_	18.70 ± 0.02a	14.96 ± 0.01a	7.07 ± 0.02f	3.70 ± 0.01cd	2.77 ± 0.02cd	71.48 ± 0.03i
*T*_7_	17.41 ± 0.01d	12.63 ± 0.02d	8.37 ± 0.01a	3.88 ± 0.02ab	2.91 ± 0.02a	72.20 ± 0.04h
*T*_8_	16.86 ± 0.02f	11.64 ± 0.02f	6.04 ± 0.01i	3.60 ± 0.03ef	2.61 ± 0.02f	76.10 ± 0.06d
*T*_9_	16.18 ± 0.01i	9.30 ± 0.02i	7.38 ± 0.02d	3.79 ± 0. 02bc	2.75 ± 0.01de	76.77 ± 0.02c

Means with different letters in each column differ highly significantly *P* < 0.01*: dry weight basis.

**Table 6:** Regression coefficients for the models representing as a function of variations in the independent variables for hardness (firmness) of date bars and their respective *R* ^2^.

Terms of model equations	Days						
	0	15	30	45	60	75	90
Constant	115.34	468.85	−204.7	31.03	−1022.0	−150.2	−564.9
*X*	76.45	64.90	234.2	259.22	503.0	447.7	531.0
*Y*	87.03	182.37	535.8*	561.53	890.0	607.1	870.2
*X* × *X*	−6.46	−9.55	−20.3	−22.47	−41.0	−33.9	−41.5
*Y* × *Y*	−12.29	−28.92	−61.7*	−72.79	−105.0	−72.8	−110.2
*X* × *Y*	−2.45	7.08	−7.8	−4.50	−6.0	−9.9	4.4
*R* ^2^	90.6%	59.3%	85.6%	60.7%	63.3%	54.9%	57.6%

**Table 7:** Regression coefficients for the models representing as a function of variations in the independent variables for fracturability of date bars and their respective *R* ^2^.

Terms of model equations	Days						
	0	15	30	45	60	75	90
Constant	70.5340**	69.6997**	69.2231**	70.1477**	71.0520**	72.5183**	74.7418**
*X*	0.0908	0.4879*	0.7661	0.5701*	0.5857*	0.4703*	0.3155*
*Y*	0.0816	0.7057*	1.2051	1.0154*	0.9146*	0.5800*	0.3175
*X* × *X*	−0.0008	−0.0340*	−0.0622	−0.0332*	−0.0349*	−0.0281*	−0.0349*
*Y* × *Y*	0.0070	−0.0741*	−0.1312	−0.0922*	−0.0736*	−0.0350	−0.0342
*X* × *Y*	−0.0062	0.0067	0.0075	−0.0167	−0.0271	−0.0254	0.0167
*R* ^2^	93.9%	96.5%	81.8%	96.4%	94.8%	93.7%	95.1%

*significant (*P* < 0.05); **highly significant (*P* < 0.01) *X*: whey protein concentrate *Y*: vetch protein isolate.

**Table 8:** Regression coefficients for the models representing as a function of variations in the independent variables for taste of date bars and their respective *R* ^2^.

Terms of model equations	Day						
	0	15	30	45	60	75	90
Constant	4.6671***	3.8671***	3.9293***	3.1233**	3.6379***	3.6232***	3.1879***
*X*	0.3809*	0.4360**	0.3691***	0.3743	0.2760**	0.2617*	0.3010*
*Y*	0.9118**	0.9915***	1.0390***	1.2077**	0.7514**	0.6640**	0.6015**
*X* × *X*	−0.0277*	−0.0243**	−0.0264***	−0.0250	−0.0208**	−0.0257**	−0.0208*
*Y* × *Y*	−0.0875**	−0.0921**	−0.1094***	−0.1250**	−0.0843**	−0.0890**	−0.0593**
*X* × *Y*	−0.0166	−0.0291*	−0.0167**	−0.0208	0.0000	0.0166	−0.0083
*R* ^2^	89.9%	95.0%	98.8%	89.4%	95.2%	94.2%	89.6%

*significant (*P* < 0.10); **significant (*P* < 0.05); ***significant (*P* < 0.01)

*X*: whey protein concentrate

*Y*: vetch protein isolate.

**Table 9:** Cost analysis of date bars.

Ingredients	Rate (Rs./Kg)	Quantity (g/100 g bar)	Quantity (g/25 g bar)	Ingredient cost (Rs.)
Dates	120	70	17.50	2.10
Peanuts	140	6.25	1.56	0.20
Almonds	320	6.25	1.56	0.50
Composite flour	50	12.5	3.13	0.15
Other ingredients	250	5	1.25	0.30
Packing material	0.20/bar	—	1	0.20
Over head charges (10%)				0.35

Per bar (25 g)	Total cost (100 Kcal)			3.80

1 date bar (25 g) = Rs 3.80. PKR

1 US Dollar = 88Rs. = 23 date bars.
